# Novel, Anti-hTNF-α Variable New Antigen Receptor Formats with Enhanced Neutralizing Potency and Multifunctionality, Generated for Therapeutic Development

**DOI:** 10.3389/fimmu.2017.01780

**Published:** 2017-12-22

**Authors:** Obinna C. Ubah, John Steven, Marina Kovaleva, Laura Ferguson, Charlotte Barelle, Andrew J. R. Porter, Caroline J. Barelle

**Affiliations:** ^1^Elasmogen Ltd., Aberdeen, Scotland; ^2^Scottish Biologics Facility, Institute of Medical Sciences, University of Aberdeen, Aberdeen, Scotland

**Keywords:** variable new antigen receptor, tumor necrosis factor-α, phage display, cytokine neutralization, chronic inflammation, shark IgNAR, bi-paratopic/bi-specific binding domain, anti-TNF biologics

## Abstract

The management of chronic inflammatory diseases, such as inflammatory bowel disease, psoriasis, and rheumatoid arthritis has significantly improved over the last decade with the clinical availability of anti-TNF-α biologics. Despite this undoubted treatment success, a combination of acquired resistance together with an increased risk of systemic complications, means that a significant number of patients either fail to find a suitable targeted therapy or frustratingly discover that an approach that did work is no longer efficacious. Here, we report the isolation and characterization of a new class of super-neutralizing anti-TNF-α biologics formats, the building blocks of which were originally derived as variable new antigen receptor (VNAR) domains from an immunized nurse shark. These parental small, stable VNAR monomers recognize and neutralize tumor necrosis factor (TNF)-α, in cell-based assays, at nanomolar concentrations. However, the simple, single-chain molecular architecture of VNARs allows for easy and multiple reformatting options. Through reformatting, we achieved a 50,000-fold enhancement in *in vitro* efficacy with super-neutralizing fusion proteins able to block TNF-α induced cytotoxicity in the 2–5 pM range while retaining other functionality through the addition of fusion proteins known to extend serum half-life *in vivo*. In an *in vitro* intestinal epithelial barrier dysfunction efficacy model, the lead VNAR domains, restored barrier function and prevented paracellular flux with comparable efficacy to adalimumab (Humira^®^). In addition, all multivalent VNAR constructs restored trans-epithelial electrical resistance (TEER) to approximately 94% of the untreated control. Reformatted VNAR domains should be considered as a new class of biologic agents for the treatment of hTNF-α driven diseases; either used systemically with appropriate half-life extension or alternatively where site-specific delivery of small and stable neutralizers may provide improvements to current therapy options.

## Introduction

Tumor necrosis factor (TNF) alpha is an important cytokine produced by macrophages and a key component of the host’s defenses. TNF is released rapidly following all types of trauma and stimuli; however, excessive or persistent production often results in immunopathology, including autoimmune disease and debilitating inflammation (1, 2). It is now well established clinically that targeted neutralization of human (h)TNF-α, or blockade of the receptor mediated signaling pathway, can result in disease control and/or remission (3, 4) with a number of approved antibody-based biologics targeting either TNF-α, or their corresponding receptors in the clinic (3, 5–7). In fact, anti-TNF-α biologic approaches have, for a number of years, been the most commercially lucrative area of disease treatment for many pharmaceutical companies with the anti-TNF-α antibody adalimumab (Humira^®^) selling over $10 B every year since 2014 (2, 8, 9). Despite this success there still remain significant gaps in the available panel of anti-TNF therapies. From the very first use of anti-TNF-α biologics, clinicians have reported patient cases of non-responders or patients that become suddenly recalcitrant to first one and then subsequent anti-TNF-α biologics. Most cases of therapeutic failure are linked to the development of neutralizing anti-drug antibodies (ADA) with molecular structure, posttranslational modifications, route and frequency of administration, and duration of treatment largely associated with the development of ADAs and treatment withdrawal (10, 11). Furthermore, there is a growing body of evidence warning against the long-term exposure to systemic anti-TNF-α therapy. It appears to increase the risk of some patients developing secondary and life-threatening infections and malignancies (12–14). Therefore, there remains a need for new anti-TNF-α modalities and for new anti-TNF-α biologic formats that may avoid or at least, limit these risks. Extending therapy options for the growing number of patients who have exhausted current biologic-based anti-TNF-α control is also another desirable.

At approximately 11 kDa, variable new antigen receptor (VNAR) domains are the smallest naturally occurring independent binding domains in the vertebrate kingdom (15–21). The characteristic protruding paratopes, of VNARs means that they are often referred to as “canyon-binders,” pre-disposed to inserting themselves into pockets or grooves in proteins, resulting in an increased selection propensity of potent neutralizers of receptors and/or enzymes (22–24).

Their simple, single-domain architecture makes reformatting of these domains relatively straightforward with VNARs amenable to both *N*- and *C*- terminal molecular fusions without loss of function (21, 25–27). Here, we demonstrate that a range of additional functionalities can be added that go beyond target specificity and include: quadra-valency, bi-valent-bi-specificity, serum half-life extension, or immune system recruitment (or all four together) and that this multifunctionality can be achieved using a single fusion protein of around 50 kDa or less. While “systemic-friendly” formulations can be readily cloned and expressed, we have also shown that smaller, bi-valent, bi-specific, or even bi-paratopic formats of around 25 kDa, can be expressed cost-efficiently and at scale in non-mammalian systems. These highly stable formats (28–30) retain the neutralizing potency of their larger cousins, but are ideally suited to novel site-specific or topical administration thus minimizing the risk of the systemic side-effects often associated with parenteral administration of, for example, anti-TNF-α biologics.

In this particular study, we have isolated two anti-hTNF-α neutralizing VNARs through shark immunization and phage display and reformatted these domains as multifunctional, multivalent constructs. These fusion proteins retained their inherent binding specificity and stability while delivering improved binding affinity and super-neutralizing anti-hTNF-α potency.

## Materials and Methods

### Shark Immunization

Nurse sharks (*Ginglymostoma cirratum*) were immunized with recombinant hTNF-α (200 μg)/shark emulsified in complete Freund’s adjuvant as described by Kovaleva et al. (31). Four weeks later, 200 µg hTNF-α/shark emulsified in incomplete Freund’s adjuvant was administered. Two immunization boosts at concentrations of 100 µg hTNF-α/shark and a final boost of 50 µg hTNF-α/shark were given at 4-week intervals intravenously into the caudal vein as soluble antigen in phosphate buffered saline (PBS) (sample 0.45 µM sterile filtered). Blood samples were collected at weeks 0 (pre-immunization bleed), 10, 14, and 18. Peripheral blood lymphocytes (PBLs) isolated and total RNA prepared.

### Detection of hTNF-α Specific IgNAR in Shark Serum

IgNAR titer in the bleeds was measured using hTNF-α-coated ELISA plate. Detection was carried out using the IgNAR-specific monoclonal antibody (GA8) at a dilution of 1:200 in PBS, pH 7.4; see Müller et al. (26) for a detailed protocol.

### Total RNA Isolation from PBLs and Immune Phage Library Construction

Peripheral blood lymphocytes were harvested from the plasma of the bleed with the best IgNAR response (Bleed 5) and total RNA prepared. Total RNA from the harvested PBLs was used at approximately 2 µg/µl as template for cDNA synthesis using Superscript III First strand synthesis supermix (Invitrogen). cDNA was generated with the framework specific primers NARF4For1 (5′-ATA ATC AAG CTT GCG GCC GCA TTC ACA GTC ACG ACA GTG CCA CCT C-3′) and NARF4For2 (5′-ATA ATC AAG CTT GCG GCC GCA TTC ACA GTC ACG GCA GTG CCA TCT C-3′) (16). Following cDNA synthesis, the common framework one specific primer NARF1Rev (5′-ATA ATA AGG AAT TCC ATG GCT CGA GTG GAC CAA ACA CCG-3′) was introduced and IgNAR V (VNAR) region DNA amplified using a 3-step PCR amplification protocol. The resultant PCR product of approximately 400 base pairs was run on 1.5% agarose gel, and VNAR region cut out and purified (QIAquick purification kit, QIAGEN). Purified DNA was digested at the primer-encoded restriction sites (underlined) with the restriction enzymes *NcoI* and *NotI* (NEB), and re-purified. The VNAR restriction-enzyme digested DNA library was cloned into a pHEN2 phagemid vector (32), and transformed into a suitable *E. coli* strain.

### Phage Display Selection and Screening

A single aliquot of library stock equivalent to OD_600_ of 0.1 was added to 2× TY growth media containing 2% glucose (w/v), 100 µg/ml ampicillin, and grown at 37°C to mid-log phase (OD_600_ of 0.4–0.6) prior to infection with M13K07 helper phage (NEB). Library expression was conducted overnight in 2× TY media, 100 µg/ml ampicillin, and 50 µg/ml kanamycin at 30°C. Phage were PEG-precipitated from the culture supernatant and used for bio-panning. The library was panned against biotinylated rhTNF-α captured on streptavidin beads (Dynabeads, Invitrogen). Library phage and Dynabeads M-280 streptavidin were pre-blocked with block solution [3% (w/v) milk, 1% (w/v) BSA in PBS] for 1 h, rotating at room temperature. Biotin-rhTNF-α (400 nM) was added to blocked beads and incubated for 1 h, rotating at room temperature. Phage were deselected by incubating with blocked beads, 1 h rotating at room temperature. Biotin-rhTNF-α decorated beads were incubated with deselected phage for 1 h, rotating at room temperature. Beads were washed 5× PBST and 5× PBS prior to a strict 8-min elution with 400 µl of 100 mM Triethylamine, and neutralized by adding 200 µl of 1 M Tris–HCl pH 7.5. Mid-log phase *E. coli* TG1 cells (10 ml) were infected with 400 µl eluted phage for 30 min, at 37°C. Then, grown overnight at 37°C on TYE agar plates containing 2% glucose (w/v), 100 µg/ml ampicillin. Three further rounds of selection were conducted and stringency was increased in round 3 and 4 by reducing the concentration of biotin-rhTNF-α to 200 nM. Enrichment of antigen binding monoclonal phage was evaluated using hTNF-α-coated ELISA plates.

### VNAR Binding ELISA

Ninety-six well flat bottom Maxisorp Nunc Immuo plates (Thermo Scientific) were coated with antigen of interest [1 µg/ml hTNF-α, BSA, human serum albumin (HSA), etc.] for 1 h at 37°C or 4°C overnight. The plates were washed three times with 200 µl/well PBST [PBS with 0.1% (v/v) Tween 20] before blocking with 200 µl of 4% m (w/v) PBS (MPBS) per well and incubated at 37°C for 1 h. The blocked plates were washed three times with PBST, and 100 µl of VNAR protein solution was added per designated well and plates were incubated at room temperature for 1 h. Plates were washed three times with PBST and 100 µl of 1 in 1,000 dilution HRP conjugated anti-c-myc, anti-poly-histidine, or goat anti-human IgG antibody was added to the plates and incubated for 1 h at room temperature. The plates were washed and developed by adding 100 µl tetramethylbenzidine substrate solution and neutralized using 50 µl 1 M H_2_SO_4_.

### Determination of Anti-hTNF-α VNAR Specificity

Binding specificity was determined on ELISA plates coated with either 1 µg/ml biotin-hTNF-α or unbiotinylated hTNF-α, 10 µg/ml HSA, BSA, streptavidin, single stranded DNA, thyroglobulin, or lysozyme. ELISA plates were suitably blocked in 4% (w/v) Milk-PBS, and VNAR protein samples loaded at a top concentration of 1 µg/ml and a two-fold dilution series performed. Binding was detected with an anti-c-myc-HRP conjugated monoclonal antibody.

### Construction of Multivalent Bi-Paratopic/Bivalent VNAR Domains

For this purpose, total DNA was isolated from single clones harboring VNAR fragments of interest. Oligonucleotides required for this formatting were designed in-house, and produced by Sigma-Aldrich, UK. VNAR fragments were PCR amplified with corresponding oligonucleotide pair, thereby introducing desired restriction sites and a flexible linker (Gly_4_Ser)_n_. For dimer constructs, two VNAR fragments PCR formatted with unique cloning sites were cloned into pET28b (+) plasmid vector in two steps *via* their unique restriction sites.

For the trimeric construct, a GeneArt Gene Synthesis plasmid containing a custom-made plug-and-play DNA fragment with VNAR BA11 (a humanized anti-HSA) fragment located at base position 436–744 and flanked by both N- and C- terminal (Gly_4_Ser)_4_ flexible linkers, and cloning sites at positions 52–363 (*Xba*I/*Bam*HI) and 817–1125 (*ApaI*/*EcoR*I) for the insertion of anti-TNF VNAR fragments was utilized. Complete anti-TNF VNAR trimeric construct DNA ligated into pET28b(+) *via Xba*I and *EcoR*I restriction sites, followed by transformation into electrocompetent SHuffle^®^ T7 Express cells.

Single *E. coli* clones were picked and grown in terrific broth containing the selection antibiotic.

The Quad™ constructs were designed in-house and the gene made by GeneArt Gene Synthesis (Thermo Fisher Scientific) incorporating cloning sites *BssHII* and *EcoRI*. All Quad™ constructs were designed to incorporate a GlySer-rich short linker before linking to the hinge of a wild-type human IgG1. Specific to the Quad-X™, a (Gly_4_Ser)_4_ flexible was incorporated at the end of CH-3 and a VNAR fused to the flexible linker.

### Soluble VNAR Protein Expression and Purification

Soluble VNAR protein was expressed in both prokaryotic (*E. coli*) and eukaryotic systems (*P. pastoris*, HEK293, and CHO K1 cells). Expression in *E. coli* HB2151 cells was induced with 1 mM isopropyl β-d-1-thiogalactopyranoside (IPTG), and soluble VNAR protein was extracted from the periplasm (*E. coli* HB2151 cells) (31). All multivalent non-Fc VNAR constructs were expressed as cytoplasmic protein in IPTG-induced *E. coli* SHuffle^®^ T7 Express cells (NEB) using pET28b(+) expression vector. Extraction of cytoplasmic VNAR protein was achieved using the BugBuster™ protein extraction reagent plus Benzonase^®^ (Novagen).

Polyethylenimine-mediated transfection and transient expression in HEK293 host cells was performed using serum-free FreeStyle™ 293 media (Invitrogen) (31, 33–36). The Quad-X™ construct was also efficiently expressed in suspension-adapted CHO K1 cells by Evitria AG, Zurich, Switzerland (www.evitria.com), using an Evitria expression vector system. The cell-seed was grown in eviGrow medium, a chemically defined, animal-component free, serum-free medium. Cells were transfected with eviFect, Evitria’s custom-made, proprietary transfection reagent, and cells grown after transfection in eviMake^2^, an animal-component free, serum-free medium.

Heterologous expression of multivalent VNAR constructs in *P. pastoris* was performed by Novoprotein Scientific Inc., USA, essentially as described by Weidner et al. (36).

All VNAR constructs were purified *via* poly-histidine tag using immobilized metal affinity chromatography, while Protein-A affinity chromatography was adopted for the VNAR-Fc purification. VNAR protein eluted from affinity columns was dialyzed against PBS, pH 7.4. Electrophoresis of purified protein samples was performed on NuPAGE 4–12% Bis-Tris gels using a MES buffer system (Invitrogen) in accordance with the manufacturer’s instructions. Expression levels ranged from about 10 mg/l (non-Fc-based VNAR constructs) in *E. coli* systems to up to 150 mg/l (Fc-based VNAR constructs) in mammalian systems.

### VNAR Affinity Determination

Octet FortéBio^®^ Biolayer interferometry (BLI) was used to determine the equilibrium dissociation constant (*K*_D_). Dip and Read™ streptavidin biosensors loaded with biotinylated hTNF-α and anti-TNF-α VNAR proteins were diluted using a twofold dilution series with top concentration of 100 nM while VNAR negative controls were assayed at top concentrations of 100 nM and 1 µM. Binding association was monitored for 10 min followed by a 5 min dissociation time. The biosensor was regenerated with 2 × 300 s washes in high salt buffer, TBST (10 mM Tris, 140 mM NaCl, 0.01% Tween 20, pH 7.4).

In addition to the Octet affinity data, a single cycle kinetics screen of the VNAR constructs was also conducted using surface plasmon resonance in a BIAcore™ T200 instrument. In summary, hTNF-α was covalently bound to a CM5 sensor chip surface *via* amine coupling until an increase of around 200 response units was reached. Start-up cycles were composed of a 60-s buffer injection at a flow rate of 30 µl/min followed by a 30-s dissociation period. Anti-hTNF-α VNAR cycles consisted of a 120-s sample injection at 30 µl/min, with a 1,200-s dissociation time. A regeneration step of a 60-s injection of 10 mM glycine buffer, pH 2.0 at 30 µl/ml, followed by a 120-s stabilization period was incorporated at the end of each cycle.

### pH Stability Assessment

Variable new antigen receptor protein samples were prepared and incubated at a working concentration of 10 µg/ml in a final volume of 50 µl at designated pH value. Incubation pH 3.0 was titrated using 1 M HCl or 0.1 M citric acid against PBS, pH 7.4. VNAR protein samples were incubated at pH 3 for 28 days, at room temperature. Samples withdrawn at stipulated time points were immediately neutralized in 10× PBS, pH 7.4 to a final concentration of 0.5 µg/ml. Neutralized samples were assessed for activity retention using hTNF-α-coated ELISA plates.

### *In Vitro* hTNF-α Neutralization Assay in L929 Cells

The TNF-α sensitive mouse fibrosarcoma cell line (L929 cells) were grown to 90% confluence, seeded onto 96-well flat bottom microtiter cell culture plate at 5,000 cells per well, and incubated for 48 h. Cells were treated with 1 µg/ml actinomycin-D, before adding a 20 min co-incubated 0.3 ng/ml (1× LD_80_ dose) hTNF-α and anti-hTNF-α VNAR proteins (this step of co-incubation is not crucial as TNF-α can be added directed to wells containing cells, anti-TNF-α VNAR and actinomycin-D). Treated cells were incubated for 24 h at 37°C with 5% (v/v) CO_2_ and humidity. Cytotoxicity or cell survival was determined by adding tetrazolium salt (WST-1) cell proliferation reagent (Roche), and incubated for a further 24 h. Absorbance was read at 450 nm using a microplate reader.

### FITC-Dextran Paracellular Flux across Polarized Monolayer of Caco-2 Cells

Human epithelial colorectal adenocarcinoma cells (Caco-2) were grown to 90% confluence before seeding onto 24 wells, 0.4 µm semi-permeable tissue culture transwell inserts (Corning Inc.) at 5,000 cells per transwell insert in a final volume of 100 µl, with 600 µl complete DMEM without cells was transferred into the outer containing wells. Transwell plates were incubated at 37°C with 5% (v/v) CO_2_, and humidity, and spent DMEM + 10% (v/v) FBS replaced every 48 h. Cell proliferation was monitored under a phase contrast microscope (40× magnification objective) until cells attain 100% confluence, usually between 5 and 7 days post-seeding. Cells were grown for a further 21 days allowing differentiation, with spent medium changed every 48 h until differentiation. Designated insert wells were treated with 10 ng/ml hTNF-α, IFN-γ, and LPS with or without anti-hTNF-α VNAR proteins. Treated cells were incubated for 18 h at 37°C with 5% (v/v) CO_2_, and humidity. Following incubation for 18 h with cytokines ± anti-TNF-α VNARs, phase contrast images of treated cells were captured followed by the addition of 5 µl of 10 mg/ml fluorescein isothiocyanate-labeled dextran, molecular weight (3–5 kDa) to the apical side (insert wells) of Caco-2 monolayer. Medium from the basolateral side of the transwell chamber was collected at 2 and 24 h after addition of FITC-dextran. Fluorescence intensity was measured using a Synergy HT (BioTek^®^) microplate reader at 485 nm excitation and 520 nm emission wavelengths (37, 38).

### Epithelial Resistance Dysfunction Assay in Polarized Caco-2 Cell Monolayer

The protocol described for FITC-Dextran paracellular flux across polarized monolayer of Caco-2 cells was followed until designated cells were treated with 10 ng/ml hTNF-α, IFN-γ and LPS with or without anti-TNF-α VNAR proteins. Following incubation for 24 h with cytokines ± anti-hTNF-α VNAR domains, trans-epithelial electrical resistance (TEER) was measured in the apical chamber using Millicell^®^ ERS-2 Epithelial (Volt/Ohm) meter and MERSSTX01 electrode (Merck Millipore). Measured resistance values were normalized to the surface area under treatment.

It is important to note that 12-well tissue culture transwell inserts were seeded with 5 × 10^6^ cells/well containing 500 µl DMEM with outer well (basolateral side) containing 1.5 ml DMEM. Also during TEER measurement, DMEM volume in the insert wells was increased to 500 µl to allow volt-ohm meter electrodes to fully submerge in the medium without touching the base of the wells (37, 38).

### Statistical Analysis

Graph Pad Prism^®^, version 5.04 was used to perform statistical analysis on experiments with a minimum of three (3) independent repeat experiments with duplicates/experiment. All values shown are means of *n* = 3 experiments ± SEM (unless otherwise stated).

## Results

### Immunized Phage Library Construction

An antigen-driven IgNAR immune response was determined post immunization by measuring the IgNAR titer in the sera (pre- and post-bleeds, respectively). An increase in hTNF-α specific IgNAR was observed following each successive immunization boost up to and including the final boost. Unlike the antibody titers seen for a mouse or rabbit immunization which could be up to 100,000, the IgNAR titers appears much more slowly than an any antibody response and peaked here after five boosts at around 1,000 (results not shown).

The VNAR repertoire was PCR amplified from isolated PBLs and cloned into a phagemid vector containing an in-frame M13 bacteriophage truncated coat protein pIII gene. Library quality control including PCR amplification of insert frequency and DNA sequencing of the IgNAR V regions of a representative sample (randomly selected 300 clones) was performed. This analysis confirmed that 85% of the library incorporated a VNAR sequence, and that 79% of the library encoded functional inserts, each possessing a unique amino acid sequence in the CDR3 (data not shown). The corrected library size generated was estimated at 8.7 × 10^8^ transformants.

### Isolation of Anti-hTNF-α-Specific VNARs

Variable new antigen receptor domains specific for hTNF-α were isolated following four rounds of selection against biotinylated hTNF-α captured on streptavidin beads. Unlike solid-surface immobilized antigen, this approach presents the target in a “solution state” thereby allowing access to the antigen’s entire surface, thus maximizing the chance of isolating specific and high-affinity anti-hTNF-α VNAR. Stringency was introduced in the third and fourth rounds of selection by reducing the concentration of antigen by half. As specific binding to hTNF-α was our key anticipated outcome, randomly selected output monoclonal phage from each selection were screened for binding to target antigen and unrelated non-target protein (HSA and streptavidin). A steady increase in antigen binding was observed from pre-selected clones through to round 2 (100% positive), with a drop in the number of monoclonal phage binders after round 3 (75%) and 4 (80%). No binding to unrelated non-target proteins was observed.

### Characterization of Anti-hTNF-α VNARs As Monomers

From an original panel of 24 unique (by DNA sequence) anti-hTNF-α binding clones, two VNAR domains D1 and C4 were eventually selected as leads following a series of assays that considered binding affinity, efficacy in cell-based bio-assays and expressibility in *E. coli*. The expression yield of these two monomeric domains ranged from 7 to 14 mg/l in SHuffle^®^ cells. To benchmark the performance of the VNAR leads, a single-domain anti-hTNF-α VHH TNF30 (39, 40) was included as an appropriate positive control. As monomers, VNARs D1 and C4 recorded a binding affinity (*K*_D_) of 47 and 73 nM (Table 1), and neutralized hTNF-α-induced cytotoxicity in L929 cells at 30 and 100 nM, respectively (Table 2).

**Table 1:** Binding kinetics of anti-hTNF-α variable new antigen receptor using Octet^®^ RED96 system.

Binding domain	*K*_on_ (M^−1^s^−1^)	*K*_off_ (s^−1^)	*K*_D_ (nM)	*K*_D_ (nM) BIAcore
D1	*3.6* × *10^5^*	*1.7* × *10*^−^*^2^*	n/a	*47.5*
C4	*1.1* × *10^6^*	*8.0* × *10*^−^*^2^*	n/a	*73.5*
TNF30	*8.2* × *10^4^*	*1.4* × *10*^−^*^3^*	n/a	*16.7*
D1–D1	5.0 × 10^5^	3.2 × 10^−4^	0.6	*n/a*
D1–C4	1.8 × 10^5^	1.1 × 10^−4^	0.6	*5.0*
D1–BA11–D1	1.9 × 10^6^	2.0 × 10^−4^	0.1	*4.0*
D1–BA11–C4	1.8 × 10^5^	4.8 × 10^−5^	0.3	*0.6*

*For all measurements, streptavidin Dip and Read™ biosensors were used to immobilize biotinylated hTNF-α. All assessed domains were loaded at a top concentration of 100 nM followed by a twofold dilution series. Values obtained from BIAcore binding analysis are shown in italics, and constructs not tested are shown as “not available” (n/a)*.

**Table 2:** Neutralization of 0.3 ng/ml (LD_80_) hTNF-α-induced cytotoxicity in L929 cells.

Binding molecule	ND_50_ (nM) (≥*n* = 3 ± SEM; except where otherwise stated)
D1	30 ± 3.5 (*n* = 2 ± SD)
C4	100 ± 0.1 (*n* = 2 ± SD)
TNF30	9.2 ± 2.1
D1–Fc	0.9 ± 0.14
C4–Fc	0.52 ± 0.2
D1–D1	7.0 ± 2.4
D1–C4	0.76 ± 0.06
Humira^®^ (adalimumab)	0.03 ± 0.009
D1–BA11–D1	0.38 ± 0.03
D1–BA11–C4	0.02 ± 0.09
D1–Fc–C4 (Quad-X™)	0.002 ± 0.0011
D1–C4–Fc (Quad-Y™)	0.005 ± 0.001 (*n* = 2 ± SD)
C4–D1–Fc (Quad-Y™)	0.012 ± 0.002 (*n* = 2 ± SD)

### Reformatting VNAR Monomers As Multivalent Constructs

**Figure 1:**
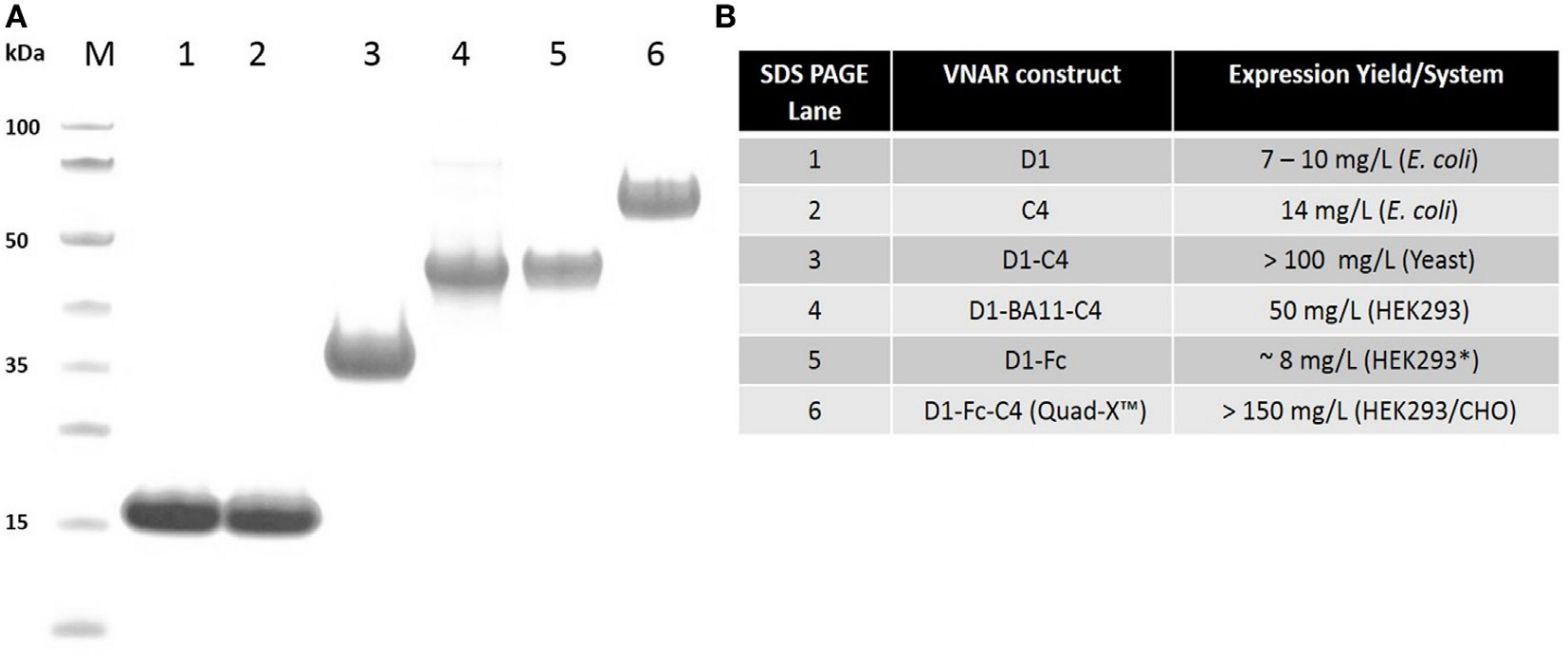
SDS PAGE and protein expression yield analysis of variable new antigen receptor (VNAR) constructs. **(A)** SDS PAGE and Coomassie blue staining of 5 µg of dithiothreitol-treated VNAR constructs. M, molecular weight marker. **(B)** Protein expression yield in specified expression systems. All expression were conducted in at least 1 l culture volume, except D1–Fc (* refers to a 200-ml culture volume).

**Figure 2:**
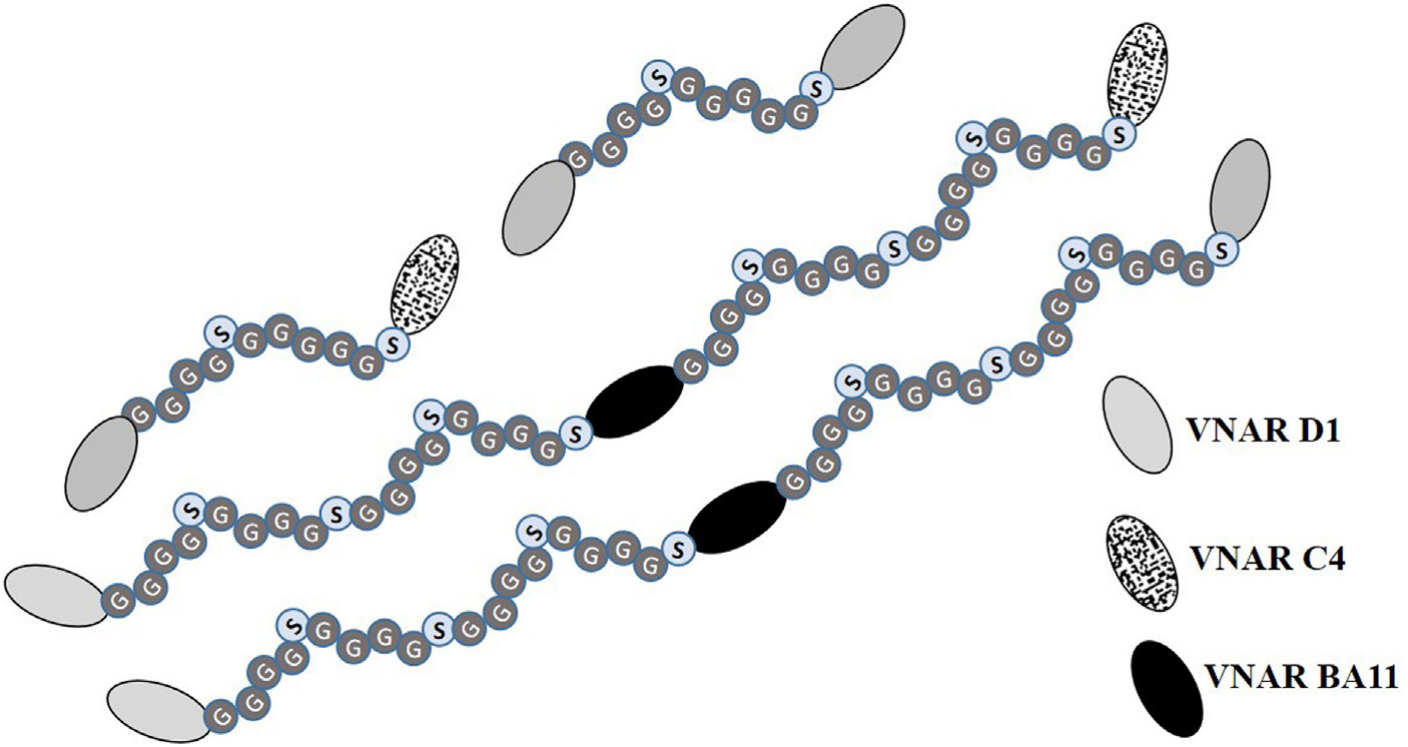
Diagrammatic representation of multivalent anti-hTNF-α variable new antigen receptor (VNAR) constructs. Bivalent constructs contain flexible (Gly_4_Ser)_2_ linkers while their trimeric counterparts in addition to having flexible GlySer linkers incorporate an anti-human serum albumin (HSA) humanized VNAR (soloMER™), called BA11 (26, 41). This additional anti-HSA binder acts as a spacer and a half-life extension tool for prolonged systemic bioavailibity of the trivalent VNAR. The functional binding of BA11 to HSA was not compromised by the presence, in the expressed fusion protein, of the anti-TNF-α binding VNARs (data not shown).

To exploit the ease with which VNARs can accommodate molecular reformatting and in an effort to significantly enhance their activity (binding affinity, avidity, and efficacy), VNARs D1 and C4 were fused *via* a flexible GlySer linker to form homo-and hetero-dimers, with a number of possible dimer outcomes (D1–D1, D1–C4, C4–C4, and C4–D1). These dimer constructs were further screened for neutralization efficacy and expressibility in *E. coli*. VNARs D1–C4 and D1–D1 showed significantly improved neutralizing potencies while expression yields remained almost unaffected (Table 2; Figure 1). In contrast, dimer constructs with VNAR C4 as the N-terminal fusion partner resulted in poor expression yields (less than 1 mg/l) and minimal efficacy improvement (data not shown). In trivalent formats, where the spacing between the functional anti-TNF-α binding domains was increased further (Figure 2) through the inclusion of a well-characterized anti-HSA binding soloMER™ (humanized VNAR) domain BA11 (41), trimers D1–BA11–D1 and D1–BA11–C4 were capable of neutralizing hTNF-α at sub-nanomolar concentrations (Table 2).

### Affinity Determination

The equilibrium dissociation constant (*K*_D_) for lead VNAR domains was determined on the Octet^®^ RED96 system as described above. As anticipated, the binding affinity of these multivalent domains significantly improved as a result of an enhanced avidity effect. The measured *K*_D_ values were in the sub-nanomolar range for the multivalent VNAR constructs, demonstrating a 600-fold improvement in affinity following reformatting of parental domains (Table 1).

### TNF-α Neutralization in L929 Cell Line

**Figure 3:**
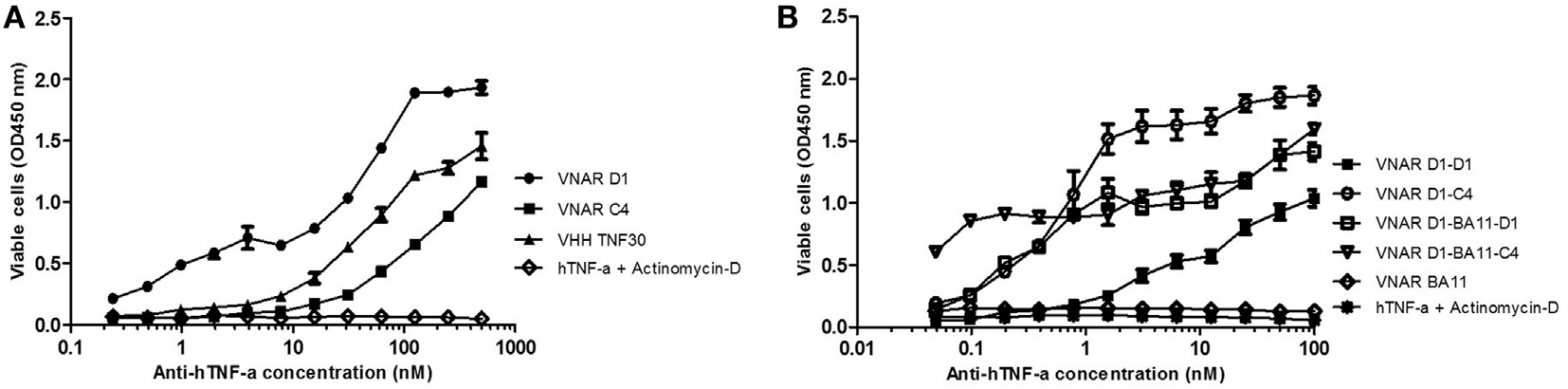
Efficacy assessment in standard L929 cell-based assay. Neutralization of 0.3 ng/ml hTNF-α-induced cytotoxicity in an actinomycin-D primed fibrosarcoma cell line (L929). **(A)** hTNF-α neutralization assay with anti-hTNF-α monomeric constructs at a maximum concentration of 500 nM. Results are the mean ± SD (*n* = 2) with two replicates per experiment. **(B)** Neutralization with multivalent anti-hTNF-α variable new antigen receptor (VNAR) constructs at maximum concentration of 100 nM. Results are the mean ± SEM (*n* = 3) with two replicates per experiment. Results **(B)** were analyzed statistically using a two-way ANOVA with Bonferroni’s multiple comparison *post hoc* test. VNAR BA11 was included as a negative control domain in all assays where D1–BA11–D1 and D1–BA11–C4 were tested.

Neutralization of hTNF-α-induced cytotoxicity in L929 cells remains one of the gold-standard *in vitro* efficacy experiments for determining the potency and therapeutic potential of anti-hTNF-α biologics. In this assay, the measured ND_50_ for the unformatted VNAR monomers D1 and C4 was ≈30 and 100 nM, respectively (Table 2). The control VHH monomer has an ND_50_ of less than 10 nM in our assay. The ability of the VNAR constructs to neutralize the cytotoxic effect of hTNF-α was significantly improved in the reformatted constructs from monomer to dimer and again to trimer with these trivalent domains showing the most enhanced potencies (Table 2). Of particular interest are the D1–C4 and D1–BA11–C4 formats. The D1–C4 had an ND_50_ value 10-fold better than its counterpart homodimer (D1–D1, ND_50_ 7 nM), and D1–BA11–C4 had an ND_50_ value of only 20 pM which is almost 20-fold better than its counterpart D1–BA11–D1 (Figure 3; Table 2). These consistent and significant improvements seen for the mixed parental dimer clones (with D1 at the N-terminal end of the fusion protein) strongly suggest that D1 and C4 bind unique epitopes on the TNF-α molecule, delivering a bi-paratopic fusion protein of improved neutralizing potency.

### Reformatting Parental Domains As VNAR-Fc Constructs

**Figure 4:**
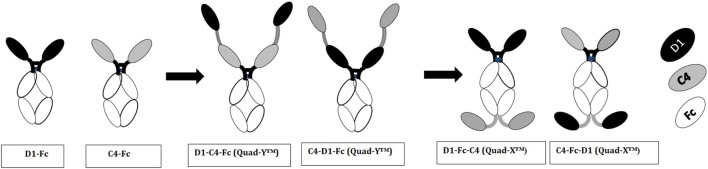
Diagrammatic representation of variable new antigen receptor (VNAR)-Fc constructs. VNAR-Fc constructs of D1 and C4 were generated by direct fusion of VNARs to the hinge of a wild-type human IgG1 Fc. These directly fused VNARs contained a Glycine-rich residue (GGGSGGGGSG) at the end of framework 4 region, and it is this glycine-rich residue that is fused to the hinge. Quad-Y™ constructs are fused to the N-terminal position of the human Fc (as shown in the diagram). The VNARs in a Quad-Y format are linked by a flexible Gly4Ser linker (GGGGSGGGGSGGGGSGGGGS). The Quad-X™ are VNARs fused to both N- and C-terminal ends of the wild-type human IgG1 Fc. The C-terminally fused VNAR is linked to the CH-3 fragment *via* a flexible Gly**_4_**Ser linker (as previously described).

**Figure 5:**
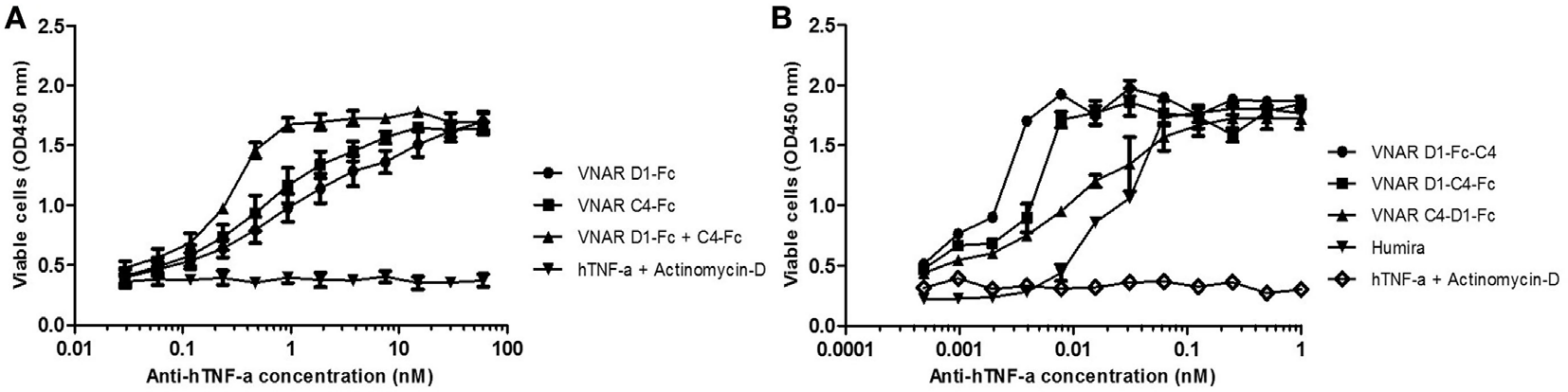
hTNF-α neutralizing capacity of the Fc-based variable new antigen receptor (VNAR) constructs. **(A)** VNAR-Fc constructs demonstrating high neutralization potencies. D1–Fc + C4–Fc denotes equimolar dosing of both constructs. Results shown are the mean ± SEM (*n* = 3) with two replicates per experiment. **(B)** A demonstration of the superior potency of the Quad™ VNAR constructs over Humira^®^ in neutralizing hTNF-α-induced cytotoxicity in L929 cells. Results shown are the mean ± SEM (*n* = 4) with two replicates per experiment (D1–Fc–C4) and mean ± SD (*n* = 2) with two replicates per experiment for the other constructs and Humira^®^.

Multivalent VNAR domains in the size range 25–40 kDa (D1–C4 and D1–BA11–C4, respectively) with picomolar neutralizing potency for hTNF-α were expressed successfully in *E. coli*. Although these small domains are well suited to site-specific delivery, they can also be administered systemically because they incorporate BA11, a half-life-extending domain (humanized VNAR), as part of the spacer region of the fusion protein (Figure 2). It is also possible to create fusion proteins that are closer to natural antibodies and incorporate an immunoglobulin Fc region. By carrying out these simple molecular fusions both N and C terminally to the human Fc region of an antibody it has been possible to generate the Quad-X™ and Quad-Y™ family of proteins both of around 50 kDa (Figure 4). When expressed in a mammalian system these proteins naturally and efficiently assemble as quadra-valent, bi-specific, and bi-paratopic proteins capable of both half-life extension and immune system recruitment. When examined in a “gold-standard” L929 assay the Quad™ family have proved to be super-sensitive neutralizers with 2–5 pM efficacy (Figure 5B) and significantly (10×) better than even the leading clinical anti-hTNF-α antibody adalimumab (Humira^®^). Even the least potent neutralizer amongst the characterized Quad™ constructs (C4–D1–Fc) is twice as potent as Humira^®^ (Table 2).

Bivalent D1–Fc and C4–Fc formats show sub-nanomolar efficacies (Figure 5A). Interestingly if equimolar dosing of both D1–Fc and C4–Fc are mixed together then a significant enhancement of efficacy is seen over the use of D1–Fc or C4–Fc alone and provides additional evidence that the two parental VNARs are likely binding different epitopes on the hTNF-α molecule (Figure 5A).

### Assessing Efficacy of Anti-hTNF-α VNARs in an *In Vitro* Model of Epithelial Barrier Dysfunction

While the Quad™ family would be suitable for parenteral administration, it is possible that the smaller dimer and trimer proteins may be ideal for topical or localized, site-specific administration avoiding the risk of systemic complications. One such clinical destination may be the lining of the gut to suppress or downregulate debilitating inflammation in conditions such as inflammatory bowel disease (IBD). While immunogenicity of VNAR domains would not be an important consideration in the intestine, their ability to tolerate and remain functional in this harsh environment would of course be vital if any therapeutic benefit is to be seen. VNARs are known to exhibit exceptional stability in harsh physiological conditions (28–30). Here, we investigated if reformatting of the parental VNAR monomers as multivalent domains impacted on this characteristic stability. In particular these studies focused on low pH 3 stability over extended periods of exposure (3–28 days). All formats tested showed an excellent tolerance to this extreme of pH even after 28 days with the dimer D1–C4 retaining almost full functionality (Table 3).

**Table 3:** Binding ELISA of variable new antigen receptor (VNAR) constructs post 28 days incubation at pH 3.

	% Retention of binding activity to hTNF-α		
Binding domain
	pH3		
	Day 3	Day 7	Day 28
D1	88 ± 10.2	51 ± 1.3	67 ± 3.4
C4	78.3 ± 3.8	85 ± 0.1	78 ± 3.8
TNF30	90 ± 0.4	78 ± 6.6	80 ± 9.7
D1–D1	91.6 ± 0.3	31.6 ± 1.6	49 ± 0.8
D1–C4	122.8 ± 0.3	106 ± 9.5	81 ± 7.4
D1–BA11–D1	97.9 ± 2.3	106 ± 1.6	77.6 ± 0.7
D1–BA11–C4	72 ± 4.2	60 ± 2.0	73.9 ± 3.8
Anti-HSAmAb	n/a	36.1 ± 0.1	1.6 (Day 21)

*VNAR protein samples incubated at pH 3 for 3–28 days were neutralized in phosphate buffered saline, pH 7.4 to a final concentration of 0.5 µg/ml before loading onto a 1 µg/ml hTNF-α and/or human serum albumin (HSA)-coated ELISA plates. Binding activity was measured as percentage residual activity compared to an untreated control at each time point. Results shown are the mean ± SD (*n* = 1 with multiple replicates per experiment). The control anti-human serum albumin monoclonal is clone HSA-11 produced in mouse (Sigma, A6684)*.

**Figure 6:**
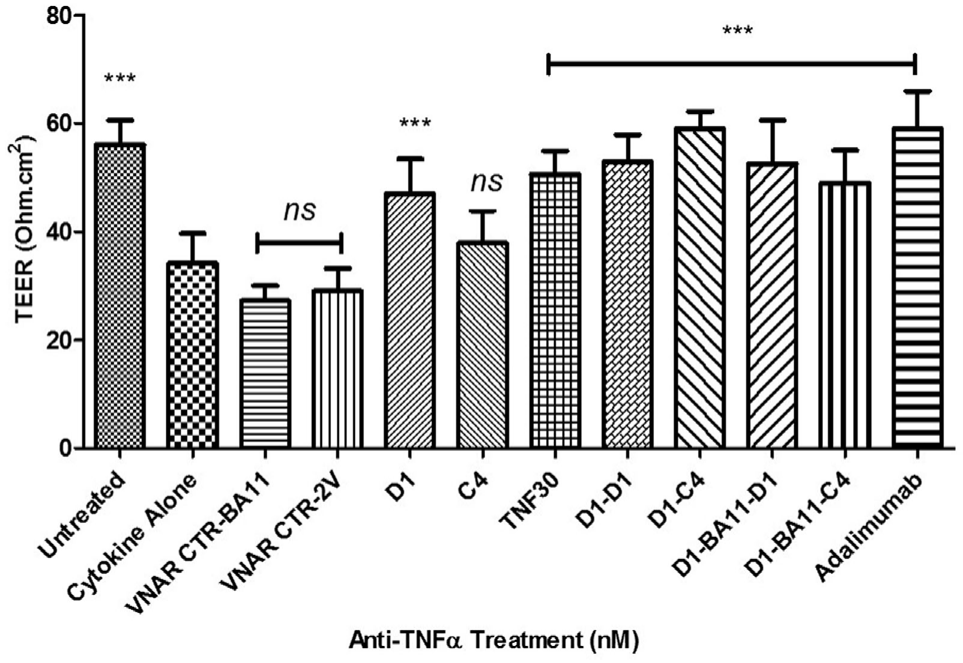
Effect of anti-hTNF-α variable new antigen receptors (VNARs) on trans-epithelial resistance disruption in Caco-2 cell model of cytokine-induced intestinal barrier dysfunction. Monolayer cells were treated with 10 ng/ml hTNF-α and IFN-γ. All VNAR monomers were added at a final concentration of 50 nM while multivalent domains and adalimumab were added at a final concentration of 25 nM. Results shown are the mean ± SD (*n* = 1 with ≥8 replicates per experiment). Results were statistically analyzed using a one-way ANOVA with Dunnett’s *post hoc* test [*** where *P* < 0.0001; not significant (ns) where *P* > 0.05]. VNAR 2V is an isotype control isolated from a naïve library, and has no inherent affinity for any known target antigen (33, 40). VNAR BA11 has been described elsewhere in this paper.

The utility of the VNAR formats was then tested in a series of bio-assays that mimic barrier dysfunction in IBD. Barrier dysfunction can be induced in IFN-γ primed Caco-2 monolayers by hTNF-α and results in disruption of tight junction morphology. Using the method described in Wang et al. (37), epithelial barrier dysfunction was created *in vitro* by treating fully differentiated Caco-2 cells with hTNF-α causing loss in barrier function (measured as leakage of solutes across tight-junctions and a decline in trans-epithelial resistance). The ability of anti-hTNF-α VNAR domains to block this dysfunction was assessed by studying both the: paracellular flux of large molecular weight hydrophilic molecules such as FITC-dextran and changes in the TEER of the treated Caco-2 cells monolayer. On average the dimer VNAR constructs restored TEER in cytokine treated cells to about 94.4% of the untreated control cells. Interestingly, VNAR D1–C4 restored TEER in cytokine treated cells to about 104.6 ± 5.34% of untreated control and was identical in potency to adalimumab (Humira^®^) which restored TEER to 103.8 ± 12.1% of untreated control (Figure 6).

**Figure 7:**
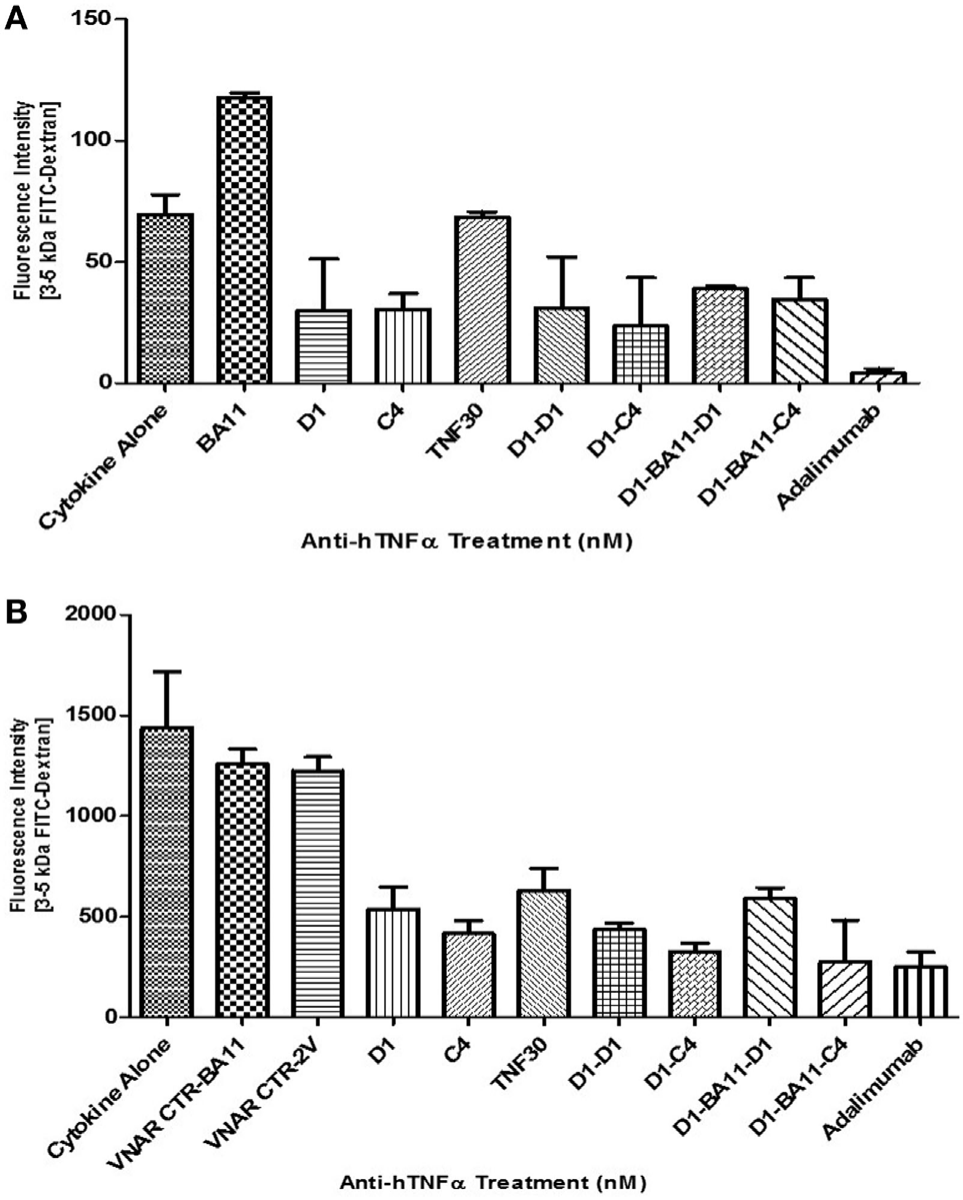
Efficacy of anti-hTNF-α variable new antigen receptors (VNARs) in preventing FITC-dextran paracellular flux in Caco-2 cell model of cytokine induced intestinal barrier dysfunction. All VNAR monomers were added at a final concentration of 50 nM while multivalent domains and adalimumab were added at a final concentration of 25 nM. FITC-Dextran flux was measured by taking media from the basolateral chamber at 2 h **(A)** and 24 h **(B)** post-addition of FITC-Dextran (i.e., 20 and 42 h post cytokine ± anti-TNF-treatment, respectively). Results shown are the mean ± SD (*n* = 1) with four replicates per experiment, and all treatment groups have been corrected for the fluorescence intensity measured in untreated cells. *y*-Axis on both bar charts are on different scales. VNAR 2V is an isotype control isolated from a naïve library, and has no inherent affinity for any known target antigen (33, 40). VNAR BA11 has been described elsewhere in this paper.

Variable new antigen receptor monomers restored barrier integrity on average to 63% of untreated, while the VNAR multivalent constructs restored integrity to about 73% of untreated control (Figure 7). VNAR D1–C4 and VNAR D1–BA11–C4 restored barrier integrity to about 78 and 84%, respectively, while adalimumab at equimolar dose restored epithelial integrity by 83%. These efficacy data clearly indicate that the VNAR multivalent constructs are comparable in *in vitro* efficacy assays. The barrier leakage can be detected after 2 h of adding FITC-dextran in the unprotected or negative control treated cells (BA11 treatment), while protection is evident even at 2 h and sustained over a 24 h period (Figure 7).

## Discussion

IgNAR V regions (VNAR) have been successfully generated against a range of targets from immunized, naïve and semi-synthetic display VNAR libraries (16, 22, 23, 26, 31, 42–45). The notion that IgNAR are part of the adaptive immune system of sharks has been reported previously (16, 26), and although time consuming, immunization still remains our preferred route for the isolation of high-affinity binders or neutralizers. Shark immunization route can be particularly useful for cross-species conserved proteins as sharks are evolutionarily distant from humans, diverging from a common ancestor approximately 450 million years ago and, therefore, the likelihood of immune tolerance to such a protein antigen is much less likely (15, 20, 46).

In our pre-selected, but immunized VNAR library, 6% of randomly selected clones were specific to hTNF-α. We also observed a post-selection preference for the characteristic CDR3-protruding type II VNARs, suggesting that our anti-hTNF-α VNARs may be accessing recessed epitopes on the hTNF-α molecule.

Here, we demonstrate extensive multivalent reformatting of VNAR fragments, with significant improvement in *in vitro* efficacy (beyond that of clinical antibodies to the same target) and multifunctionality while retaining our unique selling point of “small size” (less than 50 kDa) and flexible expressibility. Using a human antibody Fc region as a spacer in molecular fusions, we have generated for the first time super-potent quadra-valent VNAR-Fc constructs, with two main classes named Quad-X™ and Quad-Y™ after their shape when drawn in a stylized form (Figure 4). The significant improvement in binding affinity across the multivalent VNAR constructs (Table 1) is likely to be due to avidity effects. A similar stepwise trend was seen in all *in vitro* neutralization experiments, reaching a 50,000-fold efficacy improvement when comparing a single monovalent VNAR to Quad-X™ in the gold-standard L929 neutralization assay.

A number of diseases are associated with alterations in the intestinal barrier and its increased permeability. IBD, Irritable bowel syndrome, celiac disease, as well as extra-intestinal diseases such as type 1 diabetes, acquired immunodeficiency syndrome, multiple sclerosis, and rheumatoid arthritis have all been linked to intestinal barrier defects (47–51). For IBD patients that display increased intestinal paracellular permeability, TNF-α levels become elevated in the intestinal mucosa, serum, and stools of these patients (47), with recent *in vitro* studies further suggesting that increased TNF-α impairs the intestinal tight junction barrier *via* upregulation of the myosin light-chain kinase protein expression (37, 52). Existing clinically available anti-TNF therapies (e.g., adalimumab, infliximab, and certolizumab) are an effective therapy for these pateints with remission seen for Crohn’s disease and other related conditions (53). Despite the relative molecular and valency differences between the different anti-TNF-α biologic drug classes (95–150 kDa), both *in vitro* neutralization studies and clinical retrospective and non-randomized studies have demonstrated comparative efficacy (53–57). However, a common to all approach is a series of well documented systemic complications that include: immunogenicity and resulting ADA, profound issues with patient compliance because of a repeated injection dosing paradigm and serious side-effects associated with prolonged use (risk of life-threatening infections and/or lymphomas) (13, 14, 58). Therefore, the use of smaller molecular weight anti-TNF-α biologics through site-specific delivery may enhance clinical outcomes by reducing the level of systemic exposure to these powerful and pleiotropic biologic drugs (59, 60).

Intra-gastric delivery of an anti-TNF-α VHH constructs using an orally administered engineered *Lactococcus lactis* (*L. lactis*) significantly reduced inflammation in dextran sulfate sodium-induced chronic colitis mice (61). This approach effectively delivers the single-domain VHH to the colon, thereby limiting or circumventing unnecessary exposure of the domain to the very acidic pH of the gastric environment and its associated gastric enzymes. In a preliminary study, VNAR D1–C4 efficiently expressed in *L. lactis* and neutralized hTNF-α in L929 assay with ND_50_ value of 105 pM.

To partially mimic the effects of site-specific delivery, we have utilized an *in vitro* cell-based intestinal epithelial dysfunction model, and have shown that even our monovalent VNAR domains elicit excellent protection against the inflammatory toxicity generated by the presence of human TNF-α. This protection was improved when the VNARs were used in dimer or trimer formats and the level of activity seen was equivalent to that of a clinical antibody control, Humira^®^ (Figures 6 and 7). In a pH stability test, designed to mimic the environment of the gut, only the VNAR and VHH domains retained any activity (around 80%) after 7 days exposure (Table 3).

It was originally proposed, that unlike the VHH domains (62–64), VNARs might not efficiently form a dimeric fusion molecule, and even where dimerization of VNAR was achieved, the resulting dimeric construct showed a compromised expression level and binding activity thought to be due to steric hindrance or occlusion of the binding site by the incorporated linker (17). It was later shown that VNARs do in fact tolerate both N- and C-terminal molecular fusions to form either a dimer or trimer construct without significant loss of binding characteristics (25). In fact, improvements in binding kinetics, efficacy and pharmacokinetics of domains have been achieved through reformatting parental VNAR and VHH domains as multivalent and/or multi-specific domains (21, 25, 41, 63–66). Increasing the valency of binding domains such as from a monovalent Fab to a bivalent Fab fragment, monovalent scFv to bivalent scFv-Fc, or from a monovalent VHH to a bivalent, or trivalent VHH domain have shown in some cases up to a 500-fold increase in potency and/or affinity for a range of antigens and assays. Enhanced avidity and even additional mechanism of binding fragment-antigen interactions have been shown to be largely responsible for the improved efficacy seen with these multivalent binding domains (64, 65, 67). The improvements we have seen in potency is greater than one would have expected from just avidity (Tables 1 and 2) alone with some domains (e.g., D1–BA11–C4, Quad-X™ D1–Fc–C4) showing improvements of over 10,000-fold. As these jumps in potency are always associated with combinations of the D1 and C4 domains (and in that order), we postulate that some of this enhancement in neutralization may be due to bi-paratopic binding, not avidity alone. Further analysis would be required to confirm this hypothesis.

Any increase in valency of the VNAR domains increases the number of possible epitopes that can be assessed on a bioactive hTNF-α trimer, and this capacity has been optimized here using an empirically designed flexible linker (Figure 2). Previous studies have shown that multivalent constructs spaced by a short linker can achieve increased functionality by improving their antigen binding through avidity, increased antigen specificity or by cross-linking two hTNF-α trimers (64, 68, 69). We, therefore, hypothesize that the short but flexible 11 amino acid GlySer linker in the dimeric VNAR constructs (D1–C4) allow these domains to interact with two epitopes on one trimeric TNF-α molecules and/or cross-link the interaction of two epitopes on two trimeric TNF-α molecules. Similarly, the trivalent VNAR domains (D1–BA11–C4) were constructed with an increased spatial separation of the anti-TNF-α VNAR domains using two 20 amino acid flexible GlySer linkers and a 103 amino acid VNAR BA11 “spacer,” which further enhanced the ability (reach) of these constructs to interact and cross-link bioactive trimeric TNF-α molecules. The VNAR BA11, is a humanized anti-HSA soloMER™ capable of both increasing the spatial separation of the anti-hTNF-α VNAR domains and providing an option for serum half-life extension if used in a systemic, therapeutic setting (25, 41). The logical extension of this approach is our Quad-X™ family of proteins (D1–Fc–C4). Here, we have replaced the BA11 spacer with a human Fc region (half-life extension and immune system recruitment) but have enhanced the neutralizing potency of the fusion protein further by increasing the valency of both D1 and C4 domains through natural and efficient dimerization of the expressed protein. This proved to be the most potent of all the constructs tested delivering complete neutralization at only 2 pM compared to 30 pM by the world’s best-selling drug Humira^®^.

The simple molecular architecture of VNAR domains facilitates flexible reformatting options and the accommodation of additional functionality to deliver a panel of therapeutically useful formats optimized for administration both as systemic and/or site-specific drugs. This flexibility can also be used to enhance drug potency delivering a new class of biologics that can match and even surpass the activity of some of the best studied therapeutic antibodies and deliver this enhanced potency with easily expressed fusion proteins that are two-third the size of a whole antibody.

## Ethics Statement

This study was conducted in accordance with the recommendations of HMRC described in the project license—PPL 60/3799. Work conducted by the Institute of Marine and Environmental Technology (Baltimore, MD, USA) was under their ethical guidelines and authorized animal procedures. Sharks were kept in an extensive state of the art fish-holding facility as part of an environmentally responsible marine core facility that provides excellent experimental capacity for research with marine organisms. It is a completely contained, recirculating operation with large-scale mechanical and biological filtration and life support systems that enable safe and efficient re-use of tank water.

## Author Contributions

OU: scientific lead on this paper who carried out the majority of the scientific work and wrote the manuscript. JS: assisted in the rational *in-silico* construction and expression of some of the VNAR-Fc constructs. MK and LF: assisted in the library construction and expression of multivalent VNAR proteins. CB: assisted with the VNAR and monoclonal antibody stability assessment. CJB: led the science team at Elasmogen, and co-supervised this project alongside AP. Also both CJB and AP reviewed the manuscript.

## Conflict of Interest Statement

OU, JS, MK, LF, AP, and CJB are affiliated with Elasmogen Limited.

## Abbreviations

VNAR, variable new antigen receptor; TEER, trans-epithelial electrical resistance; hTNF-α, human tumor necrosis factor-alpha; IBD, inflammatory bowel disease; RA, rheumatoid arthritis; IL, interleukin; IgNAR, immunoglobulin new antigen receptor.
